# Angiogenesis in the Development of Medication-Related Osteonecrosis of the Jaws: An Overview

**DOI:** 10.3390/dj5010002

**Published:** 2016-12-26

**Authors:** Andreas Max Pabst, Maximilian Krüger, Sebastian Blatt, Thomas Ziebart, Roman Rahimi-Nedjat, Elisabeth Goetze, Christian Walter

**Affiliations:** 1 Department of Oral- and Maxillofacial Surgery, General Armed Forces Hospital, Rübenacherstr. 170, 56072 Koblenz, Germany; 2 Department of Oral- and Maxillofacial Surgery, University Medical Center Mainz, Augustusplatz 2, 55131 Mainz, Germany; maximilian.krueger@unimedizin-mainz.de (M.K.); sebastian.blatt@gmx.de (S.B.); roman.rahimi-nedjat@unimedizin-mainz.de (R.R.-N.); elisabeth.goetze@unimedizin-mainz.de (E.G.); walter@mainz-mkg.de (C.W.); 3 Department of Oral- and Maxillofacial Surgery, University Clinic, Georg-Voigt-Straße 3, 35039 Marburg, Germany; thomas.ziebart@uk-gm.de; 4 Oral and Maxillofacial Surgery, Mediplus Clinic, Haifa-Allee 20, 55128 Mainz, Germany

**Keywords:** bisphosphonate, MR-ONJ, osteonecrosis, angiogenesis, revascularization, endothelial cells, progenitor cells, microvessel sprouting

## Abstract

Medication-related osteonecrosis of the jaws (MR-ONJ) is one of the most relevant side effects of bisphosphonate therapy; it is clinically defined as a non-healing wound in combination with an avascular and necrotic jaw within ongoing bisphosphonate therapy or after completed bisphosphonate therapy. Different theories concerning the development of MR-ONJ have been reported, while the exact pathophysiology is still unknown. Recent studies have increasingly focused on angiogenesis and revascularization concerning MR-ONJ pathophysiology, which seems to be a relevant factor in the development of MR-ONJ and a possible and promising point of action for MR-ONJ prevention and therapy. Therefore, and with respect to the different aspects and specific forms of angiogenesis, the enclosed review summarizes the possible role of angiogenesis and revascularization in the pathophysiology of MR-ONJ. Special focus is given to the strong negative influence of bisphosphonates on progenitor and mature endothelial cells in vitro as well as on microvessel sprouting in vitro and in vivo, which might result in overall reduced wound healing of oral soft and hard tissues, and therefore in an exposed and avascular jaw from a clinical viewpoint. Further, it will be summarized whether and in what way the aspect of angiogenesis might be used for possible MR-ONJ prevention and therapy.

## 1. Introduction

Bisphosphonates represent one of the most important and most clinically relevant antiresorptive agents in various fields of medicine and especially oncology. These agents, which chemically resemble pyrophosphate, have revamped the therapy for a plurality of benign and malign bone diseases, such as osteoporosis, chronic osteomyelitis, osteodystrophia deformans, osteoblastic and osteolytic bone metastases of various malignancies, and malign lymphoma, e.g., multiple myeloma [1,2,3,4,5,6,7,8]. Depending on the side chains in the chemical structure, non-nitrogen-containing (e.g., clodronate) and nitrogen-containing bisphosphonates (e.g., pamidronate, zoledronate) can be distinguished, which all have different antiresorptive power compared to the reference agent etidronate (antiresorptive power = 1) [9].

Bisphosphonates’ mode of action is based on different point of actions within the mevalonate pathway, the biochemical pathway of cholesterol synthesis. Overall, toxic ATP analogues and an inhibition of the enzyme farnesylpyrophosphate-synthase lead to an inhibition of isoprenoid geranylgeraniol (GGOH) synthesis and therefore to an absence of second messenger protein prenylation of the RAS superfamily. This biochemical cascade leads to the start of apoptosis in osteoblasts and osteoclasts, ultimately leading to overall inhibited and reduced bone turnover [10].

While various side effects of bisphosphonate therapy have been known since its clinical introduction, bisphosphonate-related osteonecrosis of the jaws (BR-ONJ) was reported as a new and unpredictable side effect in 2003 for the first time, especially after pamidronate and zoledronate therapy, and continues to increase in incidence to date [11,12,13,14]. Given that necrosis development can also occur under the influence of other drugs as well, the term ‘medication-related osteonecrosis of the jaws’ (MR-ONJ) has become more and more popular in recent years [15]. For detailed information about further MR-ONJ causes, see Reference [16].

MR-ONJ is frequently observed after surgical procedures, such as tooth extractions or dental surgery. According to the guidelines of the American Association of Oral and Maxillofacial Surgeons (AAOMS), MR-ONJ is defined as avascular and necrotic bone of the maxilla and/or the mandible for at least eight weeks in combination with an inflammation of the surrounding soft tissues and a non-healing wound during or after bisphosphonate therapy. In addition, radiation in the head and neck region must be excluded in the patient’s history [15]. Further, the 2014 update of the AAOMS position paper on MR-ONJ added more detailed information concerning the aspect of antiangiogenic medications and, in general, the relevance of angiogenesis for all aspects of MR-ONJ [15].

Concerning MR-ONJ pathophysiology, different theories have been reported. One of the most popular theories describes overall reduced bone remodeling, which is induced by bisphosphonates’ strong influence on osteogenic cells (osteoblasts and osteoclasts). The resulting complete standstill of bone turnover and bone regeneration potential might consequently result in jaw necrosis after bone irritation, for example after surgical procedures, where fast, sufficient bone regeneration and healing potential are absolutely indispensible. Other popular theories describe an influence on oral soft tissues and inflammatory reactions. In this context, oral soft tissue cells, especially gingival fibroblasts and oral keratinocytes, might be directly damaged by bisphosphonates, potentially resulting in a non-healing wound or uncovered, exposed jaw bone. This process might be additionally triggered and enhanced by local inflammation, most likely caused by high local bisphosphonate concentrations in the jaw bone and the surrounding soft tissues. Interestingly, different bacteria, such as actinomycetes, have been detected in ONJ specimens, which might also have an influence on MR-ONJ development. The potential role of bacterial contamination is discussed extremely controversially in the literature since it is still unclear whether bacterial contamination is the primary reason for MR-ONJ development or simply a subsequent phenomenon of a necrotic jaw [7,17,18,19,20]. In total, over 25 different theories and enabling factors can be listed.

In addition to these theories and hypotheses, the aspects of angiogenesis, revascularization, and microvessel development concerning MR-ONJ pathophysiology have become more popular and are frequently discussed in the current literature. The role of angiogenesis might be very relevant since angiogenesis plays a fundamental key role in wound healing as well as in soft and hard tissue regeneration (oral mucosa and jaw); further, reduced angiogenesis could explain both clinical manifestations of MR-ONJ: reduced wound healing of oral soft tissues as well as avascular and necrotic bone in the context of MR-ONJ. Bisphosphonates might contact and influence blood vessel development, and thereby MR-ONJ development, in two different ways: (I) through high circulating bisphosphonate concentrations in the entire circulatory system immediately after intravenous bisphosphonate application and (II) in the region of wound healing (e.g., the oral cavity) where new blood vessels should sprout into a wound in the presence of high local bisphosphonate concentrations in the bone and the surrounding soft tissues [21].

Since angiogenesis represents a comprehensive, promising, and sufficient approach for MR-ONJ pathophysiology and new MR-ONJ therapies, this manuscript reviews all articles on in vitro and in vivo experiments concerning the topic of bisphosphonates and angiogenesis published by the authors within the time frame of 2010 to 2016. In addition, the most relevant articles concerning the aspect of angiogenesis and MR-ONJ development within the time frame of 2015 to 2016 are summarized.

## 2. Review Section

Walter et al. analyzed the influence of different nitrogen-containing (ibandronate, pamidronate, zoledronate) and non-nitrogen-containing bisphosphonates (clodronate) on human umbilical cord vein endothelial cells (HUVEC) concerning their cell viability, proliferation and migration ability as well as their cell morphology in vitro. The findings demonstrated a strong negative influence, particularly for pamidronate and zoledronate, on HUVEC cells in all assays and in the confocal laser scanning microscopy visualization, whereas the impacts of ibandronate and clodronate was less distinct. The effect of non-nitrogen-containing clodronate on HUVEC cells was particularly marginal [7]. Next, Ziebart et al. investigated the influence of different bisphosphonates (clodronate, ibandronate, pamidronate, zoledronate) on HUVEC and endothelial progenitor cells (EPC). Special attention was given to cell migration ability, microvessel sprouting ability, cell density, and the apoptosis rate of the previously mentioned progenitor and mature endothelial cells after bisphosphonate substitution in vitro. The findings of this study illustrated that HUVEC cells and EPC were significantly influenced by bisphosphonates at different concentrations compared with the non-treated control groups. Especially pamidronate and zoledronate had a strong negative influence on the tested cell lines, whereas the influence of clodronate and ibandronate was less distinct on HUVEC cells and EPC [22]. To summarize, these two in vitro studies gave evidence that MR-ONJ development may be caused by the negative influence of bisphosphonates on progenitor and mature endothelial cells—on the one hand from bisphosphonates in the circulating blood and on the other hand from bisphosphonates released from the local jaw—which might result in decreased wound healing of the soft tissues as well as in an avascular jaw.

Based on these in vitro studies, Pabst et al. designed an in vivo study and analyzed the effects of bisphosphonates on angiogenesis and microvessel sprouting in a murine 3D-matrigel assay. Matrigel plugs were subcutaneously implanted under the dorsal skin of bisphosphonate-treated (clodronate, ibandronate, pamidronate, zoledronate) nude mice and the matrigel plugs were then analyzed by CD31 immunochemistry and microvascular corrosion casting. All bisphosphonates induced a significant decrease of the microvessel density compared to the controls within the matrigel plugs, which was similarly observed for the microvessel area in the matrigel plugs. Overall, pamidronate and zoledronate had the strongest negative influence. Interestingly, ibandronate induced an increase of the mean microvessel size [23]. This study confirmed the previously mentioned in vitro results and demonstrated that especially pamidronate and zoledronate have a strong negative influence on angiogenesis and microvessel sprouting in vivo.

In further studies, the influence of geranylgeraniol (GGOH) on angiogenesis and microvessel sprouting after bisphosphonate substitution was evaluated. Ziebart et al. analyzed the influence of GGOH on cell viability, proliferation and migration ability as well as the morphological cell architecture of HUVEC cells after bisphosphonate substitution (clodronate, ibandronate, pamidronate, zoledronate) in vitro. The findings demonstrated that GGOH cell treatment was able to reverse the strong negative effects of the tested bisphosphonates on HUVEC cells and therefore angiogenesis and revascularization in vitro [24]. Based on these results, Pabst et al. investigated the effects of GGOH on EPC after bisphosphonate treatment in vitro. Cell viability, migration ability, and the apoptosis rate of EPC after bisphosphonate substitution (clodronate, ibandronate, pamidronate, zoledronate) were analyzed. All assays demonstrated strong negative effects of pamidronate and zoledronate on EPC without additional GGOH substitution. In contrast, the additional substitution of GGOH resulted in significantly increased cell viability and migration ability. Furthermore, increased EPC colony densities and decreased apoptosis rates were observed after incubation with nitrogen-containing agents [25]. A following study investigated the influence of GGOH on microvessel sprouting after bisphosphonate (BP) incubation (clodronate, ibandronate, pamidronate, zoledronate) in an in vitro 3D angiogenesis assay. For controls and clodronate, no significant differences could be detected within the experimental set-ups with or without GGOH substitution. In contrast, the strong negative influence of nitrogen-containing bisphosphonates (pamidronate and zoledronate) on microvessel sprouting could be significantly reversed by GGOH substitution [21]. To summarize, the recent studies concerning the influence of GGOH on angiogenesis, revascularization, and microvessel sprouting after bisphosphonate substitution clarified that GGOH is able to reverse bisphosphonates’ strong negative influence on angiogenesis in vitro and might be a promising candidate for MR-ONJ prevention and therapy since supportive therapeutic options for MR-ONJ are lacking.

Also, further in vitro and in vivo studies analyzed the aspect of angiogenesis in the development of MR-ONJ.

Lang et al. investigated the influence of nitrogen-containing zoledronate in different concentrations on the cell proliferation, migration ability, apoptosis rate and protein expression of vascular endothelial cells in vitro. Overall, the results demonstrated that zoledronate can significantly inhibit all the above-mentioned vascular endothelial cell characteristics and induce cell death of vascular endothelial cells and therefore affect MR-ONJ development [26]. In another in vitro study, Sharma et al. analyzed the influence of various bisphosphonates on cell differentiation of human placental mesenchymal stem cells (pMSC) of the endothelial cell lineage. The findings of this study showed that nitrogen-containing bisphosphonates were able to reduce the differentiation ability of pMSC into cells of the endothelial cell lineage which might represent a further cause or a supporting process for the development of MR-ONJ [27]. Guevarra et al. tested the effects of zoledronate on the vascular architecture in the mandible of rats after tooth extractions by using micro-CT scans. The micro-CT images showed that zoledronate-treated rats had significantly thicker, less connected and less ordered vessels compared to the control animals. These findings demonstrated that zoledronate treatment is associated with significant changes of the vascular system in the alveolar bone, which might represent a reason for MR-ONJ development [28]. Ishtiaq et al. investigated the effect of alendronate on circulating vascular endothelial growth factor (VEGF) and angiopoietin-1 (ANG-1) in post-menopausal women (T score ≤ −2) in vivo and the influence of zoledronate and alendronate on the production of VEGF and ANG-1 by osteoblasts in vitro. The results of this study led to the reasoning that bisphosphonates can suppress osteoblastic production of angiogenic factors in vitro and in vivo, which supports the relevance of angiogenesis in MR-ONJ pathophysiology [29].

In addition, the impact of zoledronate on human gingival fibroblasts and their regulation of genes associated with angiogenic regulation has been evaluated in vitro. In this study, an increased expression could be observed for RHOB, VEGF, CD55 and BMP2 genes, while CCL2 and IL6 genes were downregulated. The authors concluded that fibroblasts respond to zoledronate treatment by producing a proangiogenic environment shown by the significantly increased VEGF and BMP2 gene expressions [30]. Borke et al. analyzed the vasculature system and vascular changes in the mandibular bone of rats after bisphosphonate therapy and tooth extractions by using micro-CT visualized microvascular corrosion casts. The results demonstrated significant structural changes in the vasculature system of the mandible as well as an upregulation of VEGF as a possible result of a bisphosphonate-caused ischemia [31]. Thumbigere-Math et al. analyzed serum markers of bone turnover, angiogenesis and inflammation in BP-ONJ patients after long-term intravenous bisphosphonate therapy. However, levels of angiogenesis and inflammation markers were higher in BRONJ patients who discontinued long-term intravenous BP therapy [32]. Overall, the findings of these in vitro and in vivo studies give evidence that mechanisms exist which try to counteract and to compensate for bisphosphonates’ antiangiogenic potency.

## 3. Discussion

Overall, the current literature has demonstrated that bisphosphonates, especially the nitrogen-containing agents pamidronate and zoledronate, have a strong negative influence on angiogenesis, revascularization, and microvessel sprouting in vitro and in vivo. In addition, it could be demonstrated that the natural isoprenoid GGOH is able to reverse bisphosphonates’ strong negative influence on angiogenesis and microvessel sprouting in vitro and therefore might represent a promising and sufficient prevention and treatment option for MR-ONJ.

In this context, pamidronate and zoledronate are the agents that are most frequently associated with the development of MR-ONJ [13,14]. This is an interesting matter since nitrogen-containing ibandronate has a significantly higher antiresorptive power than nitrogen-containing pamidronate, which has a significantly stronger influence on angiogenesis and revascularization compared to ibandronate in vitro and in vivo (ibandronate = 1000, pamidronate = 100). One possible explanation for this might be the fact that both agents, ibandronate and pamidronate, significantly reduce the number of microvessels in vivo while ibandronate simultaneously increases the mean vessel diameter of the out-branching vessels. Consequently, the reduced microvessel density after bisphosphonate therapy and, therefore, the reduced blood flow might be compensated for by the increased microvessel diameter after ibandronate therapy, potentially leading to a reduced occurrence of MR-ONJ compared to pamidronate or zoledronate [23].

Some aspects concerning angiogenesis and revascularization and the development of MR-ONJ have not been evaluated to date. In this context, it should be noted that several relevant forms and manners of angiogenesis and vasculature development exist:

(I) “Angiogenesis” in general can be specified as blood vessel creation due to the development of endothelial cell bridges through the division of mature endothelial cells. In contrast, (II) “vasculogenesis” represents the formation of completely new blood vessels by endothelial progenitor cells (EPCs) which have been released from the bone marrow. (III) “Intussusceptive angiogenesis”, which is also designated as “splitting angiogenesis”, is the creation of new blood vessels by the splitting of pre-existing vessels along their longitudinal axis. This longitudinal splitting has its origin in the development of pillars [23] and enables a simple and fast increase of vessel density and overall vascularization [23]. For a long time it has been assumed that (III) intussusception has a special importance during embryonic development and is only a minor player in adults. This thesis had to be reconsidered since recent studies have demonstrated that intussusception does also have a key role in adults [21,33,34]. The current in vitro and in vivo studies strictly focused on sprouting angiogenesis from HUVEC cell spheroids in vitro or from preexisting blood vessels in murine matrigel assays in vivo [21,22,23]. The influence of bisphosphonates on special types of angiogenesis, such as intussusceptive angiogenesis, has not yet been sufficiently explored and might be an interesting topic for further studies.

With respect to new MR-ONJ prevention and therapies and the role of angiogenesis, recent studies have demonstrated that the isoprenoid GGOH can reverse the strong negative influence of especially the nitrogen-containing bisphosphonates in vitro. In vivo studies on this topic are still missing. In this context, the mode of GGOH application (topically vs. systemically) is often discussed in the literature [16,21,22,24]. The systemic application of GGOH has been reported to be dangerous because it can reduce the systemic power of the bisphosphonates in the entire organism, which can be especially critical for malignancies. In contrast, topical application seems to be reliable and should be evaluated in further studies [16,21,22,24].

Nonetheless, there is also literature available that does not underpin the importance of angiogenesis and revascularization concerning MR-ONJ development. Wehrhan et al. investigated angiogenesis and vascularization in human MR-ONJ soft-tissue specimens after zoledronate therapy and reported that vascularization and mature capillary density were unchanged. However, this study also demonstrated that vessel remodeling and microvessel formation were negatively influenced in the soft-tissue MR-ONJ specimens analyzed [16,35,36].

However, there are some aspects and findings that fortify the particular importance of angiogenesis and revascularization in MR-ONJ development [25]. It has been reported that various antiangiogenic agents, such as VEGF antibodies (Bevacizumab) and tyrosinkinase inhibitors (Sunitinib), can also cause jaw necrosis and therefore the development of MR-ONJ [37,38]. Unfortunately, it is still unclear if and how a simultaneous or time-shifted use of bisphosphonates and further antiangiogenic agents increases the risk and the extent of MR-ONJ development. In addition to the previously presented in vitro and in vivo studies concerning angiogenesis and the development of MR-ONJ, further in vivo studies have reported that the numbers of circulating progenitor and mature endothelial cells as well as VEGF serum concentrations were significantly decreased in patients after bisphosphonate therapy [25,39,40].

To conclude, the current literature deals with multiple studies demonstrating the strong influence of bisphosphonates in vitro and in vivo, giving evidence that angiogenesis and revascularization might be important aspects in MR-ONJ pathophysiology, not that they play an exclusive role—but at least an important role within the generally accepted theory of a multifactorial genesis. In addition, it could be demonstrated that GGOH is able to reverse bisphosphonates’ strong influence on angiogenesis and revascularization. Further studies in this research field should focus on (I) the influence of bisphosphonates on special types of angiogenesis, such as intussusceptive angiogenesis; and (II) whether GGOH might be able to reverse bisphosphonates’ negative influence on angiogenesis and revascularization in vivo.

As a limitation, this overview primarily summarizes the previous preclinical in vitro and in vivo studies of the presenting authors.
